# New National Geriatric 5Ms Interprofessional Core Curriculum of Geriatric Research Education and Clinical Centers

**DOI:** 10.1093/geroni/igaf122.735

**Published:** 2025-12-31

**Authors:** Kathryn Nearing, Beth Hogans, Andrea Schwartz

## Abstract

The Veterans Health Administration (VHA) is the largest provider of geriatric/gerontologic education in the US. Geriatric Research, Education, and Clinical Centers (GRECCs) are VHA centers of excellence focused on aging. Each GRECC has a health professions training program with a unique constellation of health professions trainees, ranging from chaplaincy to podiatry, pharmacy to audiology. In 2020, GRECC Associate Directors for Education and Evaluation (ADEEs) initiated the development of a common core curriculum to ensure this broad spectrum of trainees gained essential knowledge in best practices for caring for older adults. Organized by the Geriatric 5Ms, the interprofessional core curriculum was designed 1) for broad appropriateness across professions, 2) to optimize adoption by GRECC sites given diverse local contexts, and 3) to support accessibility and acceptability among trainees given expanding training requirements. This symposium will address the following topics: - Need for geriatric/gerontologic training and GRECCs’ history of training allied health professionals to promote Age Friendly Health Systems (AFHS) within the VHA and beyond - Needs assessment to inform content, which engaged subject matter experts nationally through roundtable discussions and, subsequently, ADEEs in a modified e-Delphi process - Overview of the interprofessional Geriatric 5Ms core curriculum, including its unique contribution in relation to other AFHS and Geriatric 5Ms training resources - Development of modules, delivered as micro-learning segments to optimize feasibility/utilization - Robust evaluation informed by the RE-AIM framework from implementation science. We will highlight initial evaluation findings, including emerging implementation models. The symposium will conclude with a moderated panel discussion.

